# Aortic Balloon Occlusion Technique Does Not Improve Peri-Operative Outcomes for Acute Type A Acute Aortic Dissection Patients With Lower Body Malperfusion

**DOI:** 10.3389/fcvm.2022.835896

**Published:** 2022-03-11

**Authors:** Guang Tong, Zhongchan Sun, Jinlin Wu, Shuang Zhao, Zerui Chen, Donglin Zhuang, Yaorong Liu, Yongchao Yang, Zhichao Liang, Ruixin Fan, Tucheng Sun

**Affiliations:** ^1^Department of Cardiac Surgery, Guangdong Cardiovascular Institute, Guangdong Provincial People's Hospital, Guangdong Academy of Medical Sciences, Guangzhou, China; ^2^Department of Cardiac Surgery, Ganzhou Municipal Hospital, Ganzhou, China; ^3^Department of Cardiology, Guangdong Cardiovascular Institute, Guangdong Provincial People's Hospital, Guangdong Academy of Medical Sciences, Guangzhou, China; ^4^Department of Cardiology, Ganzhou Municipal Hospital, Ganzhou, China; ^5^Department of Cardiovascular Surgery, National Center for Cardiovascular Disease, China and Fuwai Hospital, Chinese Academy of Medical Sciences and Peking Union Medical College, Beijing, China; ^6^Department of Structural Heart Disease, National Center for Cardiovascular Disease, China and Fuwai Hospital, Chinese Academy of Medical Sciences and Peking Union Medical College, Beijing, China; ^7^Key Laboratory of Cardiovascular Apparatus Innovation, Beijing, China

**Keywords:** acute type A aortic dissection, malperfusion, frozen elephant trunk, aortic arch repair, aortic balloon occlusion

## Abstract

**Background:**

The management of malperfusion is vital to improve the outcomes of surgery for acute type A acute aortic dissection (ATAAD). Open arch repair under hypothermic circulatory arrest with selective antegrade cerebral perfusion (HCA/sACP) is safe and efficient but associated with inevitable hypothermia and ischemia-reperfusion injury. The aortic balloon occlusion (ABO) technique is shown to be organ protective by allowing higher temperature and shorter circulatory arrest time. In this study, we aimed to evaluate the safety and efficacy of this new technique for ATAAD patients with lower body malperfusion.

**Methods:**

Between January 2013 and November 2020, 355 ATAAD patients with lower body malperfusion who underwent arch repair in our institute were enrolled. The patients were divided into 2 groups: ABO group (*n* = 85) and HCA/sACP group (*n* = 271). Propensity score matching was performed to correct baseline differences.

**Results:**

Using the propensity score matching, 85 pairs were generated. Circulatory arrest time was significantly lower in the ABO group compared with the HCA/sACP group (median, 8 vs. 22 min; *p* < 0.001). The incidence of in-hospital mortality (10.6 vs. 12.9%; *p* = 0.812), stroke (7.1 vs. 7.1%; *p* = 1.000), dialysis (25.9 vs. 32.9%; *p* = 0.183), hepatic dysfunction (52.9 vs. 57.6%; *p* = 0.537), tracheostomy (4.7 vs. 2.4%; *p* = 0.682), paraplegia (1.2 vs. 4.7%; *p* = 0.368) were comparable between ABO and HCA/sACP groups. Other outcomes and major adverse events were comparable. The multivariable logistic analysis did not recognize ABO technique protective against any major adverse outcomes.

**Conclusions:**

For ATAAD patients with lower body malperfusion, the ABO technique allows the performance of arch repair with frozen elephant trunk (FET) under higher temperature and shorter circulatory arrest time. However, ABO technique did not improve perioperative outcomes. Future studies are warranted to evaluate the efficacy of this technique.

## Introduction

Malperfusion is a life-threatening complication of acute type A acute aortic dissection (ATAAD). With a reported incidence of 30–40% (1, 2), it is associated with adverse post-operative outcomes (3). Malperfusion to the lower body (visceral organs, lower limbs, and spinal cord) accounts for over 70% of cases. Open-end anastomosis under hypothermic circulatory arrest in combination with selective antegrade cerebral perfusion (HCA/sACP) is the currently recommended technique for arch repair for ATAAD (4–6). The lower body is inevitably exposed to ischemia and subsequent reperfusion injury. Lower body organ injuries associated with hypothermia and ischemia-reperfusion injury remain a major concern, especially for those who already suffer from malperfusion.

By blocking the back flow with an inflated balloon inserted in the descending aorta, the aortic balloon occlusion (ABO) technique allows perfusion of the lower body *via* femoral arterial cannulation during the bulk time for open-end anastomosis, thereby shortening the lower body circulatory arrest time and permitting higher temperature. The ABO technique is shown to alleviate renal and hepatic injury in ATAAD surgery compared with the conventional HCA/sACP technique (7). The effect of the ABO technique in lower body reperfusion has never been examined. The present study discusses patients treated with the ABO technique and evaluates its efficacy in the treatment of ATAAD complicated by lower body malperfusion.

## Materials and Methods

### Patients

Between January 2013 and November 2020, a total of 1,226 patients underwent arch repair for ATAAD in our institute, and a total of 355 patients were diagnosed with lower body malperfusion at admission. Furthermore, 271 patients underwent arch repair with conventional HCA/sACP while the rest 85 patients underwent surgical repair with the ABO technique. Our study complied with the Declaration of Helsinki. The hospital's ethics committee approved the study (approval no. KY-Q-2021-073-01), and the patient consent was waived due to the study's retrospective nature.

### Definition of Lower Body Malperfusion

Lower body malperfusion was defined as compromised blood flow to a lower limb, visceral organ, spinal cord, or kidney by using a combination of clinical history, physical examination, radiographic studies, and laboratory values. Cerebral malperfusion, upper limb malperfusion, and coronary malperfusion were not considered as lower body malperfusion. For the diagnosis of lower limb malperfusion, pulse deficit, or loss of sensory or motor function of lower extremities in combination with radiographic evidence was used. Visceral malperfusion was defined as mesenteric or liver ischemia and was determined *via* a combination of clinical, laboratory, and radiographic factors, such as abdominal pain or distention, elevation of hepatic enzymes, lactic acid, and radiographic evidence of flow obstruction. Radiographic evidence of occlusion of the renal arteries or delayed enhancement with a rise in creatinine was considered evidence of renal malperfusion. Patients with transient or permanent paraplegia were considered to have spinal malperfusion. The isolated presence of a dissection flap into a branch vessel without demonstrable flow impedance by enhanced CT alone was not considered malperfusion.

### Surgical Procedure

For the ABO group, cardiopulmonary bypass (CPB) is instituted through both the right axillary and one of the femoral arteries. During the cooling phase, aortic root procedures are performed as indicated. When the nasopharyngeal temperature reaches 24–26°C, the femoral artery cannulation was clamped and the arch is longitudinally opened. The right axillary artery (RAX) was used for unilateral ACP. For bilateral ACP, both RAX and left common carotid artery (LCCA) were used. A stented graft (frozen elephant trunk, FET) (Cronus; MicroPort Scientific, Shanghai, China) was inserted into the true lumen of the descending aorta. A Foley catheter was threaded into the graft, inserted into the metal portion of the stent, and inflated with saline. Once the dilated tip of the Foley catheter is fixed against the inner wall of FET, perfusion of the lower body is resumed through the femoral artery with 2/3 of the full rate. Any back flow that does occur can be removed with suction. After the distal anastomosis was finished, the femoral arterial line was temporarily clamped. The Foley was deflated and removed, and the proximal end of the graft was clamped after de-airing. The CPB flow was gradually returned to normal and rewarming was initiated. The circulatory arrest time of the lower body was about 8 min. The ascending aorta was reconstructed during the rewarming phase.

**Figure 1:**
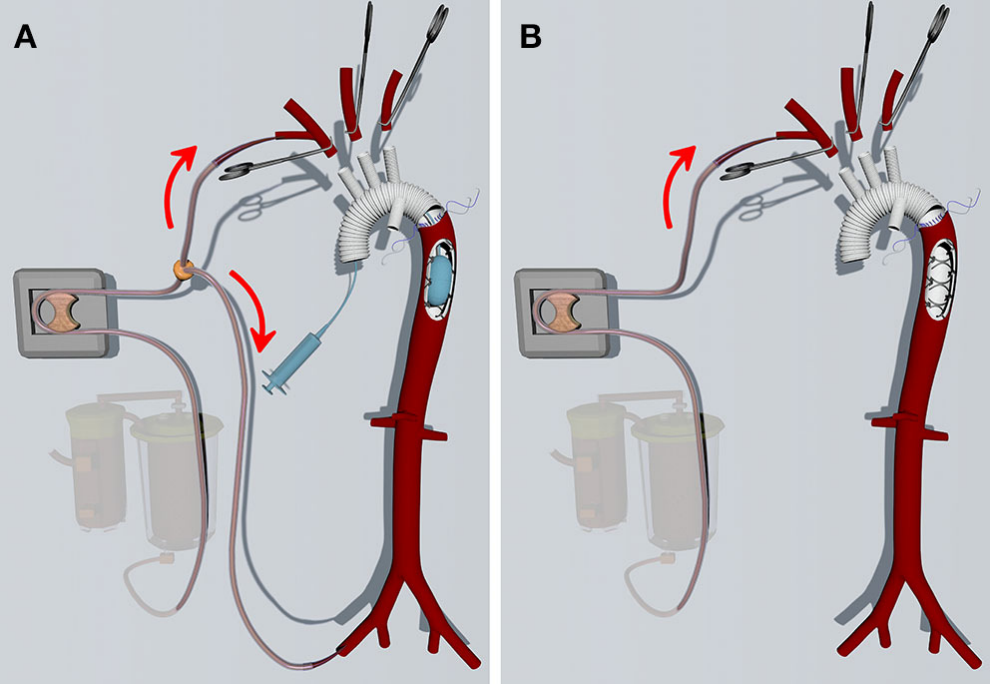
**(A)** The ABO technique. The FET is inserted into the descending aorta with selective cerebral perfusion. Then a foley tip is placed into the stented graft and inflated. Subsequently, the femoral artery cannulation is opened, and the lower body is perfused through a bifurcated arterial line when distal arch anastomosis is performed. **(B)** The conventional HCA/sACP technique. The femoral artery is not cannulated. The lower body is not perfused when the distal arch anastomosis is performed.

The HCA/sACP technique was largely based on Sun's procedure as previously described (8). The procedure differs from the ABO technique in the following ways. For most patients, the right axillary artery was cannulated for CPB and selective cerebral perfusion while the femoral artery was not cannulated. For patients unsuited for RAX cannulation, femoral artery cannulation, or central aortic cannulation under TEE guidance was used. For these patients, direct LCCA cannulation was used for unilateral ACP. ACP is instituted when the nasopharyngeal temperature reaches 22–24°C. Distal anastomosis was performed with circulatory arrest of the lower body. The circulatory arrest time was about 23 min. Proximal repair and arch branches replacement were performed as indicated (Figure 1).

### Statistical Analysis

Continuous data were evaluated for normality using the Kolmogorov–Smirnov test. The skewed data were expressed as median with interquartile range (IQR). Mann–Whitney *U*-test was used for non-normally distributed variables. Categorical variables are presented as percentages. Pearson's χ^2^ or Fisher's exact test was used for categorical variables.

To minimize the pre-operative differences between the ABO and HCA/sACP groups, a propensity score match analysis was done. A logistic regression analysis was used to calculate the propensity score for the selection of patients for the ABO and HCA/sACP groups, using pre-operative variables described in Table 1. ABO vs. HCA/sACP was the dependent variable in the logistic regression model used to compute the propensity scores. Each patient was matched to a single patient (no replacement) using the nearest neighbor matching technique and a caliper of 0.2. After matching, 85 patients for each group (ABO vs. HCA/sACP) were obtained for comparison. After matching, we assessed balance within the matched pairs using the standardized differences in covariate means. For the matched groups, means were compared using the paired Student's *t*-test and frequencies were compared using the McNemar test. Continuous variables not normally distributed were compared with Wilcoxon signed-rank test.

**Table 1:** Pre-operative characteristics.

	**Unmatched**					**Matched**				
**Variables**	**Total**	**ABO**	**HCA/sACP**	***P*** **-value**	**SMD**	**Total**	**ABO**	**HCA/sACP**	***P*** **-value**	**SMD**
	***n*** **= 356**	***n*** **= 85**	***n*** **= 271**			***n*** **= 170**	***n*** **= 85**	***n*** **= 85**		
Demographic										
Age [median (IQR)]	50.0 [43.0, 56.0]	50.0 [42.0, 58.0]	50.0 [43.0, 56.0]	0.874	0.042	50.0 [43.75, 57.0]	50.0 [42.0, 58.0]	51.0 [45.5, 57.0]	0.878	0.061
Male (%)	312 (87.6)	72 (84.7)	240 (88.6)	0.346	0.113	148 (87.1)	72(84.7)	76 (89.4)	0.361	0.140
Medical history										
Circulatory collapse (%)	16 (4.5)	1 (1.2)	15 (5.5)	0.132	0.243	3 (1.8)	1 (1.2)	2 (2.4)	1.000	0.089
Hypertension (%)	226 (63.5)	59 (69.4)	167 (61.6)	0.193	0.164	113 (66.5)	59 (69.4)	54 (63.5)	0.417	0.124
Connective tissue disorder	17 (4.8)	0 (0.0)	17 (6.3)	0.016	0.365	2 (1.2)	0 (0.0%)	2 (2.4)	0.497	0.218
Diabetes mellitus (%)	10 (2.8)	1 (1.2)	9 (3.3)	0.462	0.145	3 (1.8)	1 (1.2)	2 (2.4)	1.000	0.089
Smoking (%)	55 (15.4)	12 (14.1)	43 (15.9)	0.697	0.049	36 (21.2)	12 (14.1)	24 (28.2)	0.024	0.349
History of stroke (%)	16 (4.5)	2 (2.4)	14 (5.2)	0.376	0.148	6 (3.5)	2 (2.4)	4 (4.7)	0.682	0.127
Coronary heart disease (%)	19 (5.3)	3 (3.5)	16 (5.9)	0.581	0.112	10 (5.9)	3 (3.5)	7 (8.2)	0.329	0.200
Chronic renal dysfunction (%)	14 (3.9)	2 (2.4)	12 (4.4)	0.532	0.468	3 (1.8)	2 (2.4)	1 (1.2)	1.000	0.089
History of heart/aortic surgery (%)	8 (2.2)	0 (0.0)	8 (3.2)	0.206	0.246	0 (0.0)	0 (0.0)	0 (0.0)	–	<0.001
Atrial fibrillation (%)	1 (0.3)	0 (0.0)	1 (0.4)	1.000	0.086	0 (0.0)	0 (0.0)	0 (0.0)	–	<0.001
COPD (%)	5 (1.4)	1 (1.2)	4 (1.5)	0.026	0.026	2 (1.2)	1 (1.2)	1 (1.2)	1.000	<0.001
Malperfuison										
Cerebral (%)	19 (5.3)	6 (7.1)	13 (4.8)	0.418	0.095	9 (5.3)	6 (7.1)	3 (3.5)	0.496	0.157
Coronary (%)	84 (23.6)	1 (24.7)	3 (23.2)	0.782	0.034	42 (24.7)	21(24.7)	21(24.7)	1.000	<0.001
Renal (%)	260 (73.0)	62 (72.9)	198 (73.1)	0.982	0.003	121 (71.2)	62 (72.9)	59 (69.4)	0.611	0.078
Gastrointestinal (%)	113 (31.7)	20 (23.5)	93 (34.3)	0.062	0.239	47 (27.6)	20 (23.5)	27 (31.8)	0.230	0.184
Iliofemoral (%)	55 (15.4)	14 (16.5)	41 (15.1)	0.765	0.037	28 (16.5)	14 (16.5)	14 (16.5)	1.000	<0.001
Spinal (%)	16 (4.5)	5 (5.9)	11 (4.1)	0.479	0.084	8 (4.7)	5 (5.9)	3 (3.5)	0.7	0.111

*ABO, aortic balloon occlusion; HCA/sACP, hypothermic circulatory arrest/selective antegrade cerebral perfusion; COPD, chronic obstructive pulmonary disease*.

The multivariable logistic regression analysis was applied to determine the independent risk factors of adverse events, such as in-hospital mortality, post-operative stroke, hepatic dysfunction, dialysis, paraplegia, tracheostomy, prolonged intensive care unit (ICU) stay (>7 days), and prolonged post-operative hospital stay (>21 days). Pre-operative and intraoperative variables with a *p* < 0.1 in univariate analysis were included in the multivariable logistic regression analysis.

Statistical analyses were performed in R version 3.6.0 (R Foundation) and SPSS software v22.0 (SPSS, Inc., Chicago, IL, USA). The values of *p* < 0.05 were considered statistically significant.

## Results

### Baseline Characteristics

The baseline characteristics are summarized in Table 1. The HCA/sACP groups had more diagnosed connective tissue disorder (0.0 vs. 6.3%, *p* = 0.016). The incidences of malperfusion syndrome of the brain, heart, kidneys, lower limbs, and spinal cord were comparable in the 2 groups. The HCA/sACP group had a trend of more gastrointestinal malperfusion but the difference was not statistically significant (34.3 vs. 23.5%, *p* = 0.062). After propensity score matching, most of the pre-operative characteristics of the 2 groups (*n* = 85 in each) were comparable.

### Operative Details of Propensity Score Matching Groups

Operative details of both the full original cohort and the propensity score matched cohort are presented in Table 2. After matching, for root procedure, the Bentall operation was less used in the ABO group (24.7 vs. 43.5%, *p* = 0.010) while more commissure suspension technique was used in the ABO group (41.2 vs. 24.7%, *p* = 0.022). For distal repair, the rate of the branched graft was significantly lower in the ABO group (11.8 vs. 95.3%, *p* = 0.001) while the en-bloc technique was used for the majority of patients with ABO (88.2 vs. 4.7%, *p* = 0.001). These differences in surgical strategies were largely due to the surgeon's preference. The CPB time was comparable between the two groups (median, 241 vs. 242 min, *p* = 1.000). The ABO group demonstrated longer aortic cross-clamp time (median, 146.0 vs. 125 min, *p* = 0.001) (Supplementary Table 2) but the difference was no longer significant after matching (median, 146.0 vs. 130 min, *p* = 0.167). Circulatory arrest time was significantly shorter in the ABO group (median, 8 vs. 22 min; *p* < 0.001). The lowest temperature was significantly higher in the ABO group before (24.6 vs. 21.6°C; *p* < 0.001). Operative details of both the full original cohort are summarized in Supplementary Table 2.

**Table 2:** Operative characteristics (propensity score matching cohort).

**Variables**	**Total** **(*n* = 170)**	**ABO** **(*n* = 85)**	**HCA/sACP** **(*n* = 85)**	***P*** **-value**
Proximal repair			
Sino-tubular junction collection (%)	37 (97.6)	21 (24.7)	16 (18.8)	0.353
Commissure suspension (%)	56 (32.9)	35 (41.2)	21 (24.7)	0.022
Wheats (%)	0 (0.0)	0 (0.0)	0 (0.0)	–
Bentall (%)	58 (34.1	21 (24.7)	37 (43.5)	0.010
VSRR (%)	20 (11.8)	9 (10.6)	11 (12.9)	0.634
Concomitant procedures			
CABG (%)	11 (6.5)	5 (5.9)	6 (7.1)	1.000
MVP/MVR/TVP (%)	3 (1.8)	2 (2.4)	1 (1.2)	1.000
Distal			
branched graft (%)	91 (53.9)	10 (11.8)	81 (95.3)	0.001
En-bloc (%)	79 (46.5)	75 (88.2)	4 (4.7)	0.001
FET (%)	170 (100.0)	170 (100.0)	170 (100.0)	–
SCP			
Unilateral ACP (%)	75 (44.1)	3 (3.5)	72 (84.7)	<0.001
Bilateral ACP (%)	92 (54.1)	82 (96.5)	10 (11.8)	<0.001
RCP (%)	3 (1.8)	0 (0.0)	3 (3.5)	0.246
Time/temperature			
CPB time [median (IQR)]	241.5 [215.75, 282.0]	241 [215.5, 287.0]	242 [216.5, 278.0]	1.000
ACC time [median (IQR)]	136.0 [112.0, 164.25]	146.0 [116.0, 171.0]	130.0 [99.50, 157.5]	0.167
HCA time [median (IQR)]	15.0 [8.0, 22.0]	8.0 [7.0, 10.0]	22 [19.0, 25.0]	<0.001.
Lowest temperature [IQR]	23.4 [21.175, 24.725]	24.6 [23.2, 27.0]	21.6 [20.4, 23.6]	<0.001.

*ABO, aortic balloon occlusion; HCA, HCA/sACP, hypothermic circulatory arrest/selective antegrade cerebral perfusion; VSSR, valve-sparing root replacement; CABG, coronary artery bypass grafting; MVP/MVR/TVP, mitral valvuloplasty/mitral vitral replacement/tricuspid valvuloplasty; ACP, antegrade cerebral perfusion; RCP, retrograde cerebral perfusion; CPB, cardiopulmonary bypass; ACC, aortic cross clamp; HCA, hypothermic circulatory arrest*.

### Perioperative Outcomes of Propensity Score Matching Groups

As shown in Table 3, there were no significant differences between the ABO and HCA/sACP groups in revisiting for bleeding (11.8 vs. 4.7%; *p* = 0.161), extracorporeal membrane oxygenation (ECMO) (5.9 vs. 2.4%; *p* = 0.443), mediastinitis (1.2 vs. 4.7%; *p* = 0.368), transient neurological deficit (TND) (31.8 vs. 32.9%; *p* = 0.870), paraplegia (1.2 vs. 5.9%; *p* = 0.210), dialysis (25.9 vs. 35.3%; *p* = 0.183), hepatic dysfunction (52.9 vs. 57.6%; *p* = 0.537), tracheostomy (4.7 vs. 2.4%; *p* = 0.682), post-operative stroke (7.1 vs. 7.1%; *p* = 0.834), and in-hospital mortality (10.6 vs. 12.9%; *p* = 0.812).

**Table 3:** In-hospital outcomes (propensity score matching cohort).

**Variables**	**Total** **(*n* = 170)**	**ABO** **(*n* = 85)**	**HCA/sACP (*n* = 85)**	***P*** **-value**
Revisiting for bleeding (%)	40 (11.2)	10 (11.8)	30 (11.1)	0.860
ECMO (%)	10 (2.8)	5 (5.9)	5 (1.8)	0.063
Mediastinitis (%)	12 (3.4)	1 (1.2)	11 (4.1)	0.307
TND (%)	104 (29.2)	27 (31.8)	77 (28.4)	0.553
Paraplagia (%)	12 (3.4)	1 (1.2)	11 (4.1)	0.307
dialysis (%)	111 (31.2)	22 (25.9)	89 (32.8)	0.227
Hepatic dysfunction (%)	164 (46.1)	45 (52.9)	119 (43.9)	0.145
Tracheostomy (%)	17 (4.8)	4 (4.7)	13 (4.8)	1.000
Stroke (%)	27 (7.6)	6 (7.1)	21 (7.7)	0.834
In-hospital mortality (%)	48 (13.5)	9 (10.6)	39 (14.4)	0.370
Ventilation time, d [median (IQR)]	5.0 [2.0, 7.0]	6.0 [3.5, 9.5]	4.0 [2.0, 7.0]	0.001
ICU stay, d [median (IQR)]	9.0 [6.0, 15.0]	10.0 [7.0, 19.0]	8.0 [6.0, 13.0]	0.003
Hospital stay, d [median (IQR)]	22.5 [16.0, 33.0]	25.0 [20.0, 38.5]	21.0 [15.0, 32.0]	0.008

*ABO, aortic balloon occlusion; HCA, hypothermic circulatory arrest; ECMO, Extracorporeal membrane oxygenation; TND, transient neurological deficit; ICU, intensive care unit*.

The post-operative mechanical ventilation time was longer in the ABO group before match [6 (3.5, 9.5) vs. 4 (2.0, 7.0); *p* = 0.0001] (Supplementary Table 2) but the difference was not statistically significant [6 (3.5, 9.5) vs. 6 (2.0, 8.0); *p* = 0.0084]. ICU stay [10.0 (7.0, 19.0) vs. 9.0 (5.0, 15.0); *p* = 0.0017], hospital stay [25.0 (20.0, 38.5) vs. 21.0 (16.0, 31.0); *p* = 0.0034] were both longer in the ABO group compared with the HCA group.

### Risk Factors for Adverse Outcomes (Full Original Cohort)

The ABO technique was not an independent predictor for any adverse outcome in the multivariable regression analysis. The multivariable risk analysis for in-hospital mortality revealed no risk factors. History of stroke [*p* = 0.022; hazard ratio (*HR*), 4.216; 95% *CI*, 1.229, 14.460] was recognized as risk factor for post-operative stroke. CPB time (*p* = 0.036; *HR*, 1.006; 95% *CI*, 1.000, 1.011) was risk factor for dialysis post-operatively. In addition, the multivariable risk analysis revealed that the spinal malperfusion (*p* <0.001; *HR*, 11.803; 95% *CI*, 2.970, 46.913) was a risk factor for paraplegia. The age (*p* = 0.020; *HR*, 1.075; 95% *CI*, 1.011, 1.142) and spinal malperfusion (*p* <0.001; *HR*, 13.482; 95% *CI*, 3.404, 53.393) were risk factors for tracheostomy. The multivariable risk analysis revealed hypertension (*p* = 0.012; *HR*, 1.832; 95% *CI*, 1.142, 2.940) as a risk factor for prolonged ICU stay (>7 days). No risk factor for prolonged hospital stay (>21 days) was identified (Table 4).

**Table 4:** Multivariable logistic analysis for risk factors associated with post-operative stroke, CRRT, paraplegia, prolonged ventilation requiring tracheostomy, and prolonged ICU stay (full original cohort, *n* = 356).

**Variables**	**OR (95% CI)**	***P*** **-value**
Stroke		
History of stroke	4.216 (1.229–14.460)	0.022
Cerebral malperfusion	3.310 (0.985–11.117)	0.053
Dialysis		
Chronic renal disease	2.860 (0.937–8.725)	0.065
CPB time	1.006 (1.000–1.011)	0.036
Paraplegia		
Spinal malperfusion	11.803 (2.970–46.913)	0.001
Traecheostomy		
Age	1.075 (1.011–1.142)	0.020
Spinal malperfusion	13.482 (3.404–53.393)	0.001
Prolonged ICU stay		
HT	1.832 (1.142–2.940)	0.012

*CPB, cardiopulmonary bypass*.

## Discussion

The main findings of this study are as follows: the ABO technique allows arch repair to be performed under higher temperature and with shorter circulatory arrest time compared with conventional HCA/sACP technique. However, for patients with lower body malperfusion, the ABO technique did not reduce the incidence of adverse outcomes, such as in-hospital mortality, post-operative stroke, dialysis, liver dysfunction, paraplegia, and prolonged ventilation requiring tracheostomy.

The open-end anastomosis with systemic circulatory arrest under hypothermia has been a mainstream technique for arch repair among patients with ATAAD. The open-end anastomosis allows optimal exposure of the anastomotic site and complete visualization of the arch to rule out additional entry tears (4) and has been shown to improve both perioperative and long-term outcomes (9, 10). However, organ injuries associated with hypothermia and prolonged circulatory arrest time have been shown to be associated with adverse outcomes (11). For patients with lower body malperfusion, minimizing intraoperative ischemic injury is vital for survival.

The FET has been widely used since 2006. It expands the true lumen, improves thrombosis of the residual false lumen, and also made distal anastomosis easier to perform (12, 13). The ABO technique takes advantage of this stent placed in the descending aorta by placing an aortic balloon in it to block the backflow from femoral cannulation. This allows perfusion of the lower body during the bulk time of distal anastomosis. In the present study, the circulatory arrest time was shortened to ~8 min and allowed the lowest temperature to be raised to about 24°C.

Despite the significant reduction of circulatory arrest time and elevation of the lowest temperature, the ABO technique did not improve survival or reduce stroke. A recent analysis of 1,708 cases of aortic arch surgery found the circulatory arrest time >38 min as a risk factor for mortality and permanent neurologic dysfunction (14). The average circulatory arrest time of 22 [19.0, 25.0] min in the HCA/sACP group is safe enough and is not associated with an increased risk of mortality and stroke.

In a previous study, the ABO technique was shown to reduce liver damage and predispose to low-grade AKI by providing almost continuous blood flow to the liver and kidney compared with the HCA/sACP method (7). However, in the present study, the ABO technique did not reduce hepatic dysfunction or the need for dialysis among patients with lower body malperfusion. Malperfusion to lower body organs can be secondary to either static or dynamic obstruction (4). In dynamic obstruction, the pressurized false lumen intermittently displaces the mobile dissection flap over the orifice of the branch vessel. The dynamic obstruction can be relieved by depressurizing the false lumen and the true lumen flow can be restored by the ABO technique. In static obstruction, true or false lumen thrombosis formed distal to the pressurized false lumen and static obstruction may not be released by opening of the proximal aorta, in this case, the ABO technique would be futile to relieve malperfusion.

This study has several inherited limitations. First, the study is a retrospective review, bias and confounding may persist despite our attempts to control for them with propensity score-matching. All procedures were treated at a single center, our results may not translate to other clinical settings, as disparities exist across institutes. The number of patients in the study was relatively small. Multi-centered, large sample studies are warranted in the future.

## Conclusion

The ABO technique allows the performance of arch repair with FET under higher temperature and shorter circulatory arrest for ATAAD patients with lower body malperfusion. However, the ABO technique did not reduce the incidence of major adverse outcomes. Future large-sample, randomized, multicenter studies are warranted to thoroughly evaluate the efficacy of this technique.

## Data Availability Statement

The raw data supporting the conclusions of this article will be made available by the authors, without undue reservation.

## Ethics Statement

The studies involving human participants were reviewed and approved by Ethics Committee of the Guangdong Provincial People's Hospital. Written informed consent for participation was not required for this study in accordance with the national legislation and the institutional requirements.

## Author Contributions

RF, TS, and GT conceived the research question and conceived and designed the analysis. SZ, ZC, DZ, YL, YY, and ZL undertook data collection and conducted the study. GT, ZS, and JW drafted the manuscript. All authors reviewed the results, commented on the manuscript, and approved the final manuscript.

## Funding

This work was supported by the National Natural Science Foundation of China [81500183 to GT], the Science and Technology Program of Guangzhou, China [202002020037 to TS], the Medical Scientific Research Foundation of Guangdong Province, China [A2020011 to ZC], Guangdong Provincial People's Hospital Cardiovascular Research Fund [2020XXG004 to ZS], and the National Natural Science Foundation of Guangdong Provincial People's Hospital [KY012020289 to ZS].

## Conflict of Interest

The authors declare that the research was conducted in the absence of any commercial or financial relationships that could be construed as a potential conflict of interest.

## Publisher's Note

All claims expressed in this article are solely those of the authors and do not necessarily represent those of their affiliated organizations, or those of the publisher, the editors and the reviewers. Any product that may be evaluated in this article, or claim that may be made by its manufacturer, is not guaranteed or endorsed by the publisher.
