# Supplementary material for: Aortic Balloon Occlusion Technique Does Not Improve Peri-Operative Outcomes for Acute Type A Acute Aortic Dissection Patients With Lower Body Malperfusion
Source: Front Cardiovasc Med. 2022 Mar 11;9:835896. doi: 10.3389/fcvm.2022.835896 (PMC8962400; doi:10.3389/fcvm.2022.835896)
Supplement: Supplementary file 1 [file Data_Sheet_1.docx]

Supplementary Table 1. Operative characteristics (full original cohort)

| Variables | Total (n=356) | ABO (n=85) | HCA/sACP (n=271) | P value |
| --- | --- | --- | --- | --- |
| Proximal repair |  |  |  |  |
| Sino-tubular junction collection (%) | 112 (31.5) | 21 (24.7) | 91 (33.6) | .124 |
| Commissure suspension (%) | 104 (29.2) | 35 (41.2) | 69 (25.5) | .006 |
| Wheats (%) | 5 (2.3) | 0 (0.0) | 5 (3.5) | .165 |
| Bentall (%) | 124 (34.8) | 21 (24.7) | 103 (38.0) | .025 |
| VSRR (%) | 31 (8.7) | 9 (10.6) | 22 (8.1) | .481 |
| Concomitant procedures |  |  |  |  |
| CABG (%) | 24 (6.7) | 5 (5.9) | 19 (7.0) | .810 |
| MVP/MVR/TVP (%) | 8 (2.2) | 2 (2.4) | 6 (2.2) | 1.000 |
| Distal |  |  |  |  |
| branched graft (%) | 260 (73.0) | 10 (11.8) | 250 (92.3) | .000 |
| En-bloc (%) | 96 (27.0) | 75 (88.2) | 21 (7.7) | .000 |
| FET (%) | 356 (100.0) | 85 (100.0) | 271 (100.0) | .089 |
| SCP |  |  |  |  |
| Unilateral ACP (%) | 235 (66.0) | 3 (3.5) | 232 (85.6) | .000 |
| Bilateral ACP (%) | 116 (32.6) | 82 (96.5) | 34 (12.5) | .000 |
| RCP (%) | 5 (1.4) | 0 (0.0) | 5 (1.8) | .597 |
| Time/Temperature |  |  |  |  |
| CPB time (median [IQR]) | 238.5  [209.25,279.0] | 241  [215.5, 287.0] | 238  [206.0, 275.0] | 0.384 |
| ACC time (median [IQR]) | 130  [100.0, 159.75] | 146  [116.0, 171.0] | 125  [96.0, 153.0] | 0.001 |
| HCA time (median [IQR]) | 20  [12.25, 24.75] | 8  [7, 10.0] | 22  [19.0, 26.0] | 0.001 |
| Lowest temperature [IQR]) | 22.1  [19.3, 24.1] | 24.6  [23.2, 27.0] | 21.0  [18.7, 23.6] | 0.001 |

Supplementary Table 2. Operative characteristics (full original cohort)

| Variables | Total (n=356) | ABO (n=85) | HCA/sACP (n=271) | P value |
| --- | --- | --- | --- | --- |
| Revisiting for bleeding (%) | 40 (11.2) | 10(11.8) | 30(11.1) | .860 |
| ECMO (%) | 10 (2.8) | 5(5.9) | 5(1.8) | .063 |
| Mediastinitis (%) | 12 (3.4) | 1(1.2) | 11(4.1) | .307 |
| TND (%) | 104 (29.2) | 27(31.8) | 77(28.4) | .553 |
| Paraplagia (%) | 12 (3.4) | 1(1.2) | 11(4.1) | .307 |
| dialysis (%) | 111 (31.2) | 22(25.9) | 89(32.8) | .227 |
| Hepatic dysfunction (%) | 164 (46.1) | 45 (52.9) | 119 (43.9) | .145 |
| Tracheostomy (%) | 17 (4.8) | 4(4.7) | 13(4.8) | 1.000 |
| New stroke (%) | 27 (7.6) | 6 (7.1) | 21 (7.7) | .834 |
| In-hospital Mortality (%) | 48 (13.5) | 9(10.6) | 39(14.4) | .370 |
| Ventilation time, d, (median [IQR]) | 5.0  [2.0, 7.0] | 6.0  [3.5, 9.5] | 4.0  [2.0, 7.0] | 0.001 |
| ICU stay, d, (median [IQR]) | 9.0  [6.0, 15.0] | 10.0  [7.0, 19.0] | 8.0  [6.0, 13.0] | 0.003 |
| Hospital stay, d, (median [IQR]) | 22.5  [16.0, 33.0] | 25.0  [20.0, 38.5] | 21.0  [15.0, 32.0] | 0.008 |
